# Looking inside the black box: results of a theory-based process evaluation exploring the results of a randomized controlled trial of printed educational messages to increase primary care physicians’ diabetic retinopathy referrals [Trial registration number ISRCTN72772651]

**DOI:** 10.1186/1748-5908-9-86

**Published:** 2014-08-06

**Authors:** Jeremy M Grimshaw, Justin Presseau, Jacqueline Tetroe, Martin P Eccles, Jill J Francis, Gaston Godin, Ian D Graham, Janet E Hux, Marie Johnston, France Légaré, Louise Lemyre, Nicole Robinson, Merrick Zwarenstein

**Affiliations:** Ottawa Hospital Research Institute, The Ottawa Hospital - General Campus, 501 Smyth Road, Box 711, Ottawa, Ontario K1H 8 L6 Canada; Department of Medicine, University of Ottawa, 451 Smyth Rd, Ottawa, Ontario K1H 8 M5 Canada; Institute of Health and Society, Newcastle University, Baddiley-Clark Building, Richardson Road, Newcastle Upon Tyne, NE2 4AX England; Canadian Institutes of Health Research, 160 Elgin St, Ottawa, Ontario K1A 0 W9 Canada; School of Health Sciences, City University London, Northampton Square, London, EC1V 0HB UK; Faculty of Nursing, Laval University, Pavillon Ferdinand-Vandry, 1050 Avenue de la Medicine, Room 1445, Quebec City, Quebec G1V 0A6 Canada; School of Nursing, Faculty of Health Sciences, University of Ottawa, 451 Smyth Rd, Ottawa, Ontario K1H 8 M5 Canada; Canadian Diabetes Association, 522 University Ave, Toronto, ON M5G 2A2 Canada; Institute of Applied Health Sciences, College of Life Sciences and Medicine, 2nd floor, Health Sciences Building, Foresterhill, Aberdeen, AB25 2ZD UK; Department of Family Medicine and Emergency Medicine, Université Laval, Québec City, Québec G1K 7P4 Canada; School of Psychology, 120 University, Social Sciences Building, Ottawa, Ontario K1N 6N5 Canada; Institute for Clinical Evaluative Sciences, University of Toronto, 2075 Bayview Avenue, Toronto, Ontario M4N 3M5 Canada

**Keywords:** Process evaluation, Theory of planned behavior, Printed educational material, Healthcare professional behavior, Behavior change

## Abstract

**Background:**

Theory-based process evaluations conducted alongside randomized controlled trials provide the opportunity to investigate hypothesized mechanisms of action of interventions, helping to build a cumulative knowledge base and to inform the interpretation of individual trial outcomes. Our objective was to identify the underlying causal mechanisms in a cluster randomized trial of the effectiveness of printed educational materials (PEMs) to increase referral for diabetic retinopathy screening. We hypothesized that the PEMs would increase physicians’ intention to refer patients for retinal screening by strengthening their attitude and subjective norm, but not their perceived behavioral control.

**Methods:**

Design: A theory based process evaluation alongside the Ontario Printed Educational Material (OPEM) cluster randomized trial. Postal surveys based on the Theory of Planned Behavior were sent to a random sample of trial participants two months before and six months after they received the intervention. Setting: Family physicians in Ontario, Canada. Participants: 1,512 family physicians (252 per intervention group) from the OPEM trial were invited to participate, and 31.3% (473/1512) responded at time one and time two. The final sample comprised 437 family physicians fully completing questionnaires at both time points. Main outcome measures: Primary: behavioral intention related to referring patient for retinopathy screening; secondary: attitude, subjective norm, perceived behavioral control.

**Results:**

At baseline, family physicians reported positive intention, attitude, subjective norm, and perceived behavioral control to advise patients about retinopathy screening suggesting limited opportunities for improvement in these constructs. There were no significant differences on intention, attitude, subjective norm, and perceived behavioral control following the intervention. Respondents also reported additional physician- and patient-related factors perceived to influence whether patients received retinopathy screening.

**Conclusions:**

Lack of change in the primary and secondary theory-based outcomes provides an explanation for the lack of observed effect of the main OPEM trial. High baseline levels of intention to advise patients to attend retinopathy screening suggest that post-intentional and other factors may explain gaps in care. Process evaluations based on behavioral theory can provide replicable and generalizable insights to aid interpretation of randomized controlled trials of complex interventions to change health professional behavior.

**Trial registration:**

ISRCTN72772651.

**Electronic supplementary material:**

The online version of this article (doi:10.1186/1748-5908-9-86) contains supplementary material, which is available to authorized users.

## Background

Printed educational materials (PEMs) are a commonly used mode for delivering professional behavior change interventions. A recent Cochrane review of printed educational materials identified seven randomized controlled trials (RCTs) with categorical outcomes and observed a median absolute risk difference of +2% (range 0% (deterioration of care) to +11%), and identified three RCTs with continuous outcomes, observing a median standardised mean difference of 0.13 (range from -0.16 to +0.36) [1]. However the majority of included studies were small and had methodological problems. The Ontario Printed Educational Materials (OPEM) retinopathy trial, published alongside this process evaluation, is a large trial of PEMs and demonstrated no benefit of any form or combination of PEMs (‘short’ or ‘long’ educational messages with or without patient reminder notes) in improving attendance of family physicians’ patients with diabetes for retinopathy screening—only about 30% of patients in the study received regular diabetic retinopathy screening [2]. The rigorous methods and precision of the estimated effect provided compelling evidence of the lack of any effect of the intervention within the trial, but provided no information about why the intervention was unsuccessful.

Process evaluations collect data alongside randomized trials of complex interventions to explore possible causal mechanisms and effect modifiers [3, 4]. This is akin to measuring intermediate endpoints in clinical trials to further understand the biological basis of any observed effects (for example, measuring lipid profiles alongside trials of lipid-lowering drugs where a primary endpoint could be reduction in vascular related deaths). Theory-based process evaluations offer the added advantage of using validated constructs (and measures) to explore hypothesized mechanisms of action of interventions that can help to build a cumulative knowledge base while also informing the interpretation of individual trial outcomes [5]. The OPEM trial aimed to change physicians’ behavior, and we hypothesized that the interventions would achieve this through changes in physicians’ intention, as a consequence of enhancing their positive attitude to retinal screening, and their awareness of retinal screening as normative in this situation, but the interventions would not alter physicians’ perceptions of their abilities to carry out the behavior (refer for retinopathy screening). We conducted a prospective theory-based process evaluation alongside the OPEM trial to test the hypothesis that the intervention would operate through increasing physicians’ intention to refer for retinopathy screening [5] using the Theory of Planned Behavior (TPB), a well validated social cognition model [6–8].

## Methods

### Setting

The methods of both the OPEM trial and the OPEM theory-based process evaluation have been described in detail [5, 9]. The OPEM trial intervention was replicated across three different behaviors; in this paper, we report the results of the process evaluation of the OPEM retinopathy trial. Briefly, the OPEM retinopathy trial used a 2 × 3 factorial design to test the effectiveness of three forms of printed educational materials (short or long educational messages and patient reminder notes) to enhance physicians’ referrals of patients with diabetes for retinopathy screening. Retinopathy is a common complication in diabetes and when left untreated can lead to loss of vision. Prevention can be achieved through regular retinal screening and treatment when retinopathy is detected. Screening rates in Ontario are low, with less than half of people aged between 30 and 64 years newly diagnosed with diabetes being screened [2].

The main trial report provides a detailed description of the PEM interventions [2]. Briefly, the PEMs were embedded within an existing newsletter mailed to 15,000 healthcare providers in Ontario. Physicians were randomized to receive a factorial combination of either a short bullet-point based outsert stapled to the outside of the newsletter; and/or a longer two-page insert within the newsletter. Those randomized to receive an outsert were also randomized to receive a pad of reminders that could be given to patients to remind them to make an eye exam appointment.

### Theory of planned behavior and outcome measures

**Figure 1:**
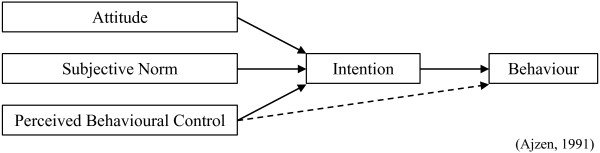
**The theory of planned behavior.**

The TPB [6] states that the proximal antecedents of behavior are intention to perform the behavior and perceived behavioral control (PBC) over the behavior. Intention is predicted by three underlying social cognition-based mechanisms: attitude (*i.e.*, in favor or not) towards the behavior in question, the influence of subjective norm (*i.e.*, perceptions of others’ views, in this instance most likely peers and experts) concerning the behavior, and their perceived control over the behavior (PBC; *i.e.*, the ease with which they could execute a referral should they wish to do so; see Figure 1). Therefore, intention was the primary outcome and attitude, subjective norm, and PBC were secondary outcomes and were believed to be prior in the causal pathway for referral.

Using standard methods [10, 11] we developed a TPB questionnaire that had 18 items, each scored with a seven point Likert scale (see Additional file 1). TPB items were reverse-scored if necessary so that high scores represented agreement (or positive attitude) and low scores, disagreement (or negative attitude). We used the mean of the items measuring each theory-based construct to create a composite score that ranged from one to seven for each construct: Intention (three items; pre- and post-test Cronbach’s α = 0.93), Attitude (five items, pre-test α = 0.92, post-test α = 0.93), Subjective Norm (five items, pre-test α = 0.92, post-test α = 0.94), PBC (four items, pre- and post-test α = 0.79).

### Study participants and sample size

Study participants were randomly chosen from the family practitioner participants of the OPEM trial by the OPEM trial research team. We required 378 participants to have 80% power of detecting an effect size of 0.5 standard deviations using a significance level of 5%, assuming a 50% response rate for each survey (pre- and post-intervention) we surveyed 1,512 family physicians.

### Procedure

In addition, we asked an open-ended question about physicians’ perceptions of factors that might influence whether patients were screened for diabetic retinopathy. We surveyed the same participants two months before and six months after the OPEM retinopathy intervention. We used Dillman’s total design method to maximize response rates [12]. In addition, we provided $20 (CDN) to every physician who returned a completed survey [13, 14].

### Analysis

Independent data entry verification was conducted for a 10% sample of responses and a data entry error rate of less than 1% was found. To compare responders and non-responders, we extracted personal and demographic data from a random sample of 20% of each group from the MD Select physician database [15].

Using analyses of covariance in SPSS, we compared groups factorially on their post-intervention TPB construct scores while adjusting for baseline differences to determine whether there had been changes in the predicted constructs across the study groups as hypothesized. We used a p-value of 0.01 to determine statistical significance to adjust for multiple testing.

The comments from the open ended questions were coded thematically by three independent coders and grouped into categories involving physicians’ beliefs about advising their patients to attend for retinal screening, and physicians’ perceptions about patients’ beliefs and actions following their advice. Discrepancies were resolved through majority decision and discussion with a fourth member of the team when required.

### Ethics approval

This study received approval from the Research Ethics Boards at The Ottawa Hospital and Sunnybrook Health Sciences Centre.

## Results

### Response rates, representativeness of respondents and reliability of the composite measures

Six hundred and forty-nine participants (of 1,512; 42.9%) responded at time one and, of these, 473 (72.9%) responded at time two (giving a cumulative response rate of 31.3%). Table 1 details the response rates. There were no significant differences between pre-intervention respondents and non-respondents (Additional file 2). Respondents who completed both the pre- and post-intervention surveys were compared to those who completed only the pre-intervention questionnaire to explore the impact of response attrition on the results. Using each group’s pre-intervention TPB scores, analysis of variance found no significant differences between groups. Thirty-six cases (36/473 = 7.6%) were excluded from analyses (listwise deletion) due to missing data. The final sample was 437 Ontario family practitioners who responded to both pre- and post-intervention surveys and had no missing data on any of the TPB variables.

**Table 1:** **Participant flow by group**

*Retinopathy screening (N=437)*			
		Insert	No insert
**Outsert**	**Patient Reminder Note**	n=252 Baseline questionnaires sent n=144 Baseline non-response	n=252 Baseline questionnaires sent n=136 Baseline non-response
		n=36 Follow-up attrition	n=34 Follow-up attrition
		n=5 Excluded	n=6 Excluded
		**n=67 Analysed**	**n=76 Analysed**
	**No Patient Reminder Note**	n=252 Baseline questionnaires sent n=150 Baseline non-response	n=252 Baseline questionnaires sent n=146 Baseline non-response
		n=20 Follow-up attrition	n=29 Follow-up attrition
		n=6 Excluded	n=4 Excluded
		**n=76 Analysed**	**n=73 Analysed**
**No Outsert**	**No Patient Reminder Note**	n=252 Baseline questionnaires sent n=141 Baseline non-response	n=252 Baseline questionnaires sent n=146 Baseline non-response
		n=33 Follow-up attrition	n=24 Follow-up attrition
		n=5 Excluded	n=10 Excluded
		**n=73 Analysed**	**n=72 Analysed**

### Assessing change in cognitions towards referring for diabetic retinopathy screening

Across all 649 respondents at baseline 580 (89.4%) physicians reported that they thought that their patients with diabetes should be screened annually or every one or two years.

Of the 437 participants providing pre- and post-intervention data, at pre-intervention, physicians reported strong intention (mean = 6.29), positive attitude (mean = 6.18), strong subjective norm (mean = 6.07) and strong perceived behavioral control (mean = 6.04) to refer the patient with diabetes for retinopathy screening (Table 2). No improvements in intention, attitude, subjective norm and perceived behavioral control were observed post intervention for any form of printed educational message (controlling for baseline scores; Table 3 for primary outcome ANCOVA results and Additional file 3 for secondary results).

**Table 2:** **Pre- and post-intervention descriptive statistics of primary and secondary outcomes by intervention group**

				Intention		Attitude		Subjective norm		PBC	
				Mean ( *SD*)		Mean ( *SD*)		Mean ( *SD*)		Mean ( *SD*)	
Experimental factors			*N*	Pre-inter	Post-inter	Pre-inter	Post-inter	Pre-inter	Post-inter	Pre-inter	Post-inter
No Insert	No Outsert	No patient reminder	72	6.32 (1.15)	6.24 (1.18)	6.15 (1.11)	6.15 (1.16)	6.14 (1.30)	6.23 (1.03)	6.14 (1.07)	6.06 (1.12)
	Outsert	No patient reminder	73	6.40 (0.95)	6.38 (1.00)	6.32 (0.81)	6.29 (0.84)	6.12 (1.07)	6.24 (0.88)	6.27 (0.97)	6.38 (0.73)
		Patient reminder	76	6.21 (1.37)	6.33 (1.12)	6.30 (0.98)	6.10 (1.18)	6.02 (1.37)	6.17 (1.21)	6.05 (1.22)	6.14 (1.07)
Insert	No Outsert	No patient reminder	73	6.10 (1.43)	6.03 (1.48)	5.92 (1.25)	6.18 (1.05)	5.78 (1.55)	5.80 (1.59)	5.87 (1.24)	6.07 (1.05)
	Outsert	No patient reminder	76	6.31 (1.23)	6.20 (1.41)	6.15 (1.03)	6.11 (1.13)	6.11 (1.18)	6.04 (1.33)	5.92 (1.27)	5.92 (1.20)
		Patient reminder	67	6.40 (1.06)	6.15 (1.53)	6.22 (0.99)	6.15 (1.16)	6.27 (1.01)	5.99 (1.40)	6.02 (1.17)	5.97 (1.29)

*Note. PBC*, Perceived Behavioral Control; *Pre-inter*, Pre-intervention survey; *Post-inter*, Post-intervention survey.

**Table 3:** **Analysis of covariance for primary outcome of change in intention**

						95% CI	
TPB Construct	Source	*df*	*F*	*p*	B	Lower	Upper
Intention	Covariate						
	Intention Pre-intervention	1	71.05	0.000	0.401	0.308	0.495
	Main Effects						
	Insert	1	2.25	0.135	-0.172	-0.398	0.054
	Outsert	1	0.53	0.469	0.102	-0.174	0.377
	Reminder Note	1	0.04	0.838	-0.029	-0.305	0.248

### Physicians’ perceptions about factors influencing diabetic retinopathy screening

Over 80% of physicians in the sample identified at least one barrier or facilitator in response to the open-ended question. Respondents identified a range of physician-level factors that influenced whether they refer patients about diabetic retinopathy screening including: their clinical assessment of the patient (‘Degree of diabetic control,’ ‘Any change in her vision’); their beliefs around when screening was required (‘No perceived need in asymptomatic patient’ , ‘I strongly recommend this for all my diabetic patients annually’); time constraints (‘Main obstacle is busy-ness of practice’); difficulty remembering to advise patients (‘Forgetting to tell patient to do so’); patient loss to follow up in general practice (‘Non-attendance at my clinic for regular diabetic tests/care’); and administrative burden associated with referral (‘I need to write referral letter to ophthalmologists every year’). Respondents also identified patient-level factors affecting screening including: patients’ perceptions of likely benefits from screening (‘Patient not convinced of importance of screening’); patients’ awareness of health insurance coverage issues (‘Sometimes people don't go because eye exams were delisted by Ontario Health Insurance Plan even though diabetics are covered’); distance to specialist services (‘Geographic location of specialists and travel requirements are the largest problems’); and waiting times for specialists appointment (‘Wait time to see specialist,’ ‘Difficulty of getting appointment with ophthalmologist’).

## Discussion

### Summary of key findings

In the face of suboptimal performance, we identified very positive cognitions of family physicians to refer their patients with diabetes for retinopathy screening during the baseline period of the OPEM retinopathy trial. There were no statistically significant increases in these cognitions following the OPEM interventions. Physicians reported both physician and patient factors that they felt might influence referral of patients for retinopathy screening.

### Interpretation and implications of results

The interventions in the OPEM trial were chosen based on empirical evidence of their potential effectiveness across a number of conditions and settings [16], rather than any specific, or theoretically driven, assessment of the determinants of retinopathy screening. The high levels of intention, and positive attitude, social norm, and PBC scores at baseline suggest that the majority of physicians already intended to perform this evidence-based behavior before the distribution of the printed educational materials. Further there was no increase in intention following the intervention. This suggests that factors other than intention and PBC at the physician level may account for the low rates of the desired behavior of patients. The physicians in the study were able to identify post-intentional factors at both the physician and patient level that may influence whether patients receive retinopathy screening. This could explain why educational materials or physician distributed patient reminders may not be effective for changing behaviors for which physicians’ intentions, perceived control, and underlying cognitions are already consistent with the evidence.

### Strengths and limitations

The strengths of this study include the use of a well-validated theory to explore proposed causal mechanisms alongside a large rigorous pragmatic trial of a professional behavior change intervention. There is increasing recognition of the value of process evaluations alongside trials of complex interventions such as professional behavior change interventions [3, 4]. Commonly process evaluations have used qualitative methods to explore participants’ attitudes towards and experiences of study interventions that provide valuable context specific information that can help interpret the results of an individual trial, but may be less helpful in predicting the likely generalizability of findings due to the lack of standardized constructs and measurements. In contrast, our theory-based process evaluation used a previously validated behavioral theory concerning determinants of behavior that enhances the generalizability and replicability of our methods. Using a behavioral theory strengthened our ability to explore the reasons underlying the failure of a complex intervention to change health professional behavior, allowing future interventions to cumulatively build on these generalizable insights. The addition of an open-ended question provided some insight to the individual and contextual variables contributing to the sub-optimal retinopathy screening rate observed in the trial.

Potential study limitations relate to the survey response rates and choice of theory. The response rates to the surveys were similar to those in other recent surveys of physicians, though may indicate some selection bias favoring more motivated respondents; however, our non-response analyses suggest no major differences in key characteristics between responders and non-responders. Whilst we were confident that the TPB included the key constructs that we believed would be influenced by the interventions, it mainly focuses on a limited number of motivational factors and does not represent all of the potential determinants of family physicians’ behavior. Future theory-based process evaluations could take a broader theoretical approach.

### Implications for research

The study has a number of implications for future research. First, this study highlights the importance of considering a broad range of potential determinants of health care professional behavior including post intentional factors. Second, it suggests that when planning intervention studies, researchers should prospectively identify factors likely to mediate the proposed mechanism of action for the behavior change and use this information to develop their intervention (in order to avoid evaluating interventions likely to be ineffective) and to identify variables which reflect the mediating processes (to enhance the informativeness of the trial). Third, theory-based process evaluations can be undertaken alongside evaluations of interventions, even where those interventions have not been developed based on an explicit behavioral theory, provided that the researchers conducting the process evaluation are able to make explicit hypotheses about the likely mediating mechanisms of the interventions and identify theories that correspond to these mechanisms. Finally, it reinforces the need to make explicit the hypothesized mechanism(s) of action for proposed professional behavior change interventions, which can be informed by the explicit use of validated theory.

## Conclusions

Lack of change in the primary and secondary theory-based outcomes provides an explanation for the lack of observed effect of the main OPEM trial. High baseline levels of intention to advise patients to attend retinopathy screening suggest that post-intentional and other factors may explain gaps in care. Process evaluations based on behavioral theory can provide replicable and generalizable insights to aid interpretation of RCTs of complex interventions to change health professional behavior.

## Electronic supplementary material

Additional file 1: **Questionnaire.** (DOC 54 KB)

Additional file 2: **Results for pre-intervention respondents and non-respondents demographic comparison.** (DOC 49 KB)

Additional file 3: **Analysis of covariance for secondary outcomes of change in attitude, subjective norm, and perceived behavioral control.** (DOCX 85 KB)
