# Supplementary material for: Looking inside the black box: results of a theory-based process evaluation exploring the results of a randomized controlled trial of printed educational messages to increase primary care physicians’ diabetic retinopathy referrals [Trial registration number ISRCTN72772651]
Source: Implement Sci. 2014 Aug 6;9:86. doi: 10.1186/1748-5908-9-86 (PMC4261878; doi:10.1186/1748-5908-9-86)
Supplement: Supplementary file 1 — Additional file 1: Questionnaire. (DOC 54 KB) [file 13012_2013_796_MOESM1_ESM.doc]

**Supplementary File 1: Questionnaire**

Mrs. Janet White has been your patient for a few years. She is visiting you today for her diabetic check up. She is 57 years old, married, with no children. She works as a clerk in a small government office. About 5 years ago, you diagnosed her with type 2 diabetes. After having tried unsuccessfully to achieve the suggested glycemic target with lifestyle change, she was put on metformin 500mg BID. She takes no other medication. In her medical file, you notice that she has maintained an adequate control of her hemoglobin A1C (< 7%) since starting metformin. In the past year, her LDL-C has been < 3.5 and her ratio TC: LDL-C has been < 5.0. She had a negative retinal screening 12 months ago. Other lab results are non contributory. She does not smoke. Her physical exam today is as follows: she has a BMI of 25, her blood pressure is 125/75 and her foot exam was normal. Home monitoring indicates that her blood glucose is adequate.

Please read each question carefully and answer it to the best of your ability. There are no correct or incorrect responses; we are merely interested in your point of view. The questionnaire may appear to be monotonous since several of the statements are worded in a repetitive manner.  It is the nature of this study that entails this methodological approach.  However, your collaboration is vital.  It is important to get *your opinion*.

I think the Canadian Diabetes Association (CDA) would approve of me advising this woman to make an appointment for retinal screening within the next 12 months.

| Strongly agree | 1 | 2 | 3 | 4 | 5 | 6 | 7 | *Strongly disagree* |
| --- | --- | --- | --- | --- | --- | --- | --- | --- |

I think most general practitioners/family physicians would approve of me advising this woman to make an appointment for retinal screening within the next 12 months.

| Strongly agree | 1 | 2 | 3 | 4 | 5 | 6 | 7 | *Strongly disagree* |
| --- | --- | --- | --- | --- | --- | --- | --- | --- |

There are factors outside my control that would prevent me from advising this woman to make an appointment for retinal screening within the next 12 months

| Strongly agree | 1 | 2 | 3 | 4 | 5 | 6 | 7 | *Strongly disagree* |
| --- | --- | --- | --- | --- | --- | --- | --- | --- |

I feel capable of advising this woman to make an appointment for retinal screening within the next 12 months

| Strongly agree | 1 | 2 | 3 | 4 | 5 | 6 | 7 | *Strongly disagree* |
| --- | --- | --- | --- | --- | --- | --- | --- | --- |

People who are important to me professionally think that I should advise this woman to make an appointment for retinal screening within the next 12 months.

| Strongly agree | 1 | 2 | 3 | 4 | 5 | 6 | 7 | *Strongly disagree* |
| --- | --- | --- | --- | --- | --- | --- | --- | --- |

I plan to advise this woman to make an appointment for retinal screening within the next 12 months.

| Strongly agree | 1 | 2 | 3 | 4 | 5 | 6 | 7 | *Strongly disagree* |
| --- | --- | --- | --- | --- | --- | --- | --- | --- |

I think the College of Family Physicians of Canada (CFPC) would approve of me advising this woman to make an appointment for retinal screening within the next 12 months.

| Strongly agree | 1 | 2 | 3 | 4 | 5 | 6 | 7 | *Strongly disagree* |
| --- | --- | --- | --- | --- | --- | --- | --- | --- |

I have complete control over whether to advise this woman to make an appointment for retinal screening within the next 12 months

| Strongly agree | 1 | 2 | 3 | 4 | 5 | 6 | 7 | *Strongly disagree* |
| --- | --- | --- | --- | --- | --- | --- | --- | --- |

I think the Ontario Medical Association (OMA) would approve of me advising this woman to make an appointment for retinal screening within the next 12 months.

| Strongly agree | 1 | 2 | 3 | 4 | 5 | 6 | 7 | *Strongly disagree* |
| --- | --- | --- | --- | --- | --- | --- | --- | --- |

I will advise this woman to make an appointment for retinal screening within the next 12 months.

| Strongly agree | 1 | 2 | 3 | 4 | 5 | 6 | 7 | *Strongly disagree* |
| --- | --- | --- | --- | --- | --- | --- | --- | --- |

For me, advising this woman to make an appointment for retinal screening within the next 12 months would be: (Please be sure to answer **each** question)

| Very easy | 1 | 2 | 3 | 4 | 5 | 6 | 7 | *Very difficult* |
| --- | --- | --- | --- | --- | --- | --- | --- | --- |

| Good practice | 1 | 2 | 3 | 4 | 5 | 6 | 7 | *Bad practice* |
| --- | --- | --- | --- | --- | --- | --- | --- | --- |

| Helpful | 1 | 2 | 3 | 4 | 5 | 6 | 7 | *Unhelpful* |
| --- | --- | --- | --- | --- | --- | --- | --- | --- |

| Appropriate | 1 | 2 | 3 | 4 | 5 | 6 | 7 | *Inappropriate* |
| --- | --- | --- | --- | --- | --- | --- | --- | --- |

| Necessary | 1 | 2 | 3 | 4 | 5 | 6 | 7 | *Unnecessary* |
| --- | --- | --- | --- | --- | --- | --- | --- | --- |

| Satisfying | 1 | 2 | 3 | 4 | 5 | 6 | 7 | *Not satisfying* |
| --- | --- | --- | --- | --- | --- | --- | --- | --- |

I am confident that I could advise this woman to make an appointment for retinal screening within the next 12 months

| Strongly agree | 1 | 2 | 3 | 4 | 5 | 6 | 7 | *Strongly disagree* |
| --- | --- | --- | --- | --- | --- | --- | --- | --- |

I intend to advise this woman to make an appointment for retinal screening within the next 12 months.

| Strongly agree | 1 | 2 | 3 | 4 | 5 | 6 | 7 | *Strongly disagree* |
| --- | --- | --- | --- | --- | --- | --- | --- | --- |

Thinking about your last 10 diabetic patients who had not had retinal screening in the previous two years, how many of them did you advise to make an appointment for screening? ____ of 10

In your view, how often should your diabetic patients be screened for retinopathy?

❑ annually ❑ every one to two years ❑ every two years ❑ other ____________________

In your practice, who normally is responsible for your patients being screened for retinopathy?

❑ I am ❑ the patient ❑ the optometrist ❑ the ophthalmologist ❑ other _______________

In your practice, who can you rely on to ensure that your patients are screened for retinopathy?

❑ me ❑ the patient ❑ the optometrist ❑ the ophthalmologist ❑ other ______________

Please comment on factors that you think will influence (in a positive or negative way) whether or not this patient would be screened for diabetic retinopathy in the next 12 months.

________________________________________________________________________________________________________________________________________________________________________________________________________________________________________________________________________________________________________________________________________________________

______________________________________________________________________________________

**Thank you for your time**

Please fax back to **XXX XXX-XXXX** or mail, using the stamped self addressed envelope included in this package.

**Please note**: *If you do not wish to complete the questionnaire please send a blank one back to us so that we do not continue to send you reminder notices.*
