# Supplementary material for: Looking inside the black box: results of a theory-based process evaluation exploring the results of a randomized controlled trial of printed educational messages to increase primary care physicians’ diabetic retinopathy referrals [Trial registration number ISRCTN72772651]
Source: Implement Sci. 2014 Aug 6;9:86. doi: 10.1186/1748-5908-9-86 (PMC4261878; doi:10.1186/1748-5908-9-86)
Supplement: Supplementary file 2 — Additional file 2: Results for pre-intervention respondents and non-respondents demographic comparison. (DOC 49 KB) [file 13012_2013_796_MOESM2_ESM.doc]

**Supplementary File 2**

Results for pre-intervention respondents and non-respondents demographic comparison

| Demographic Factor |  | Respondents | Non-respondents | Population^1^ | Test Results^2^ |  |
| --- | --- | --- | --- | --- | --- | --- |
| Graduating year | *Mean* | 1978.22 | 1978.06 | 1980.69 | *t*(259) = .13, *p*=.90 |  |
| Gender | *Male* | 81.9% | 74.6% | 63.0% | *χ*^2^(1, N = 261) = 2.02, *p*=.16 |  |
| Urban/rural | *Urban* | 89.8% | 85.1% | 91.9% | *χ*^2^(1, N = 261) = 1.30, *p*=.26 |  |
| University affiliation | *Yes* | 10.2% | 6.7% | 9.3% | *χ*^2^(1, N = 261) = 1.05, *p*=.31 |  |
| CFPC member | *Yes* | 44.1% | 35.8% | 46.7% | *χ*^2^(1, N = 261) = 1.86, *p*=.17 |  |
| *Note.* 20% random sample. n=127 for respondents & n=134 for non-respondents [2 (respondents) & 5 (non-respondents) participants could not be found using MD Select and are thus not included in the analysis]. | | | | | |  |
| ^1^ Population based upon all physicians in Ontario specializing in either family medicine or physician/general practice (N=10429) | | | | | | |
| ^2^ Test results compare respondents to non-respondents. | | | | | | |
