# Supplementary material for: Looking inside the black box: results of a theory-based process evaluation exploring the results of a randomized controlled trial of printed educational messages to increase primary care physicians’ diabetic retinopathy referrals [Trial registration number ISRCTN72772651]
Source: Implement Sci. 2014 Aug 6;9:86. doi: 10.1186/1748-5908-9-86 (PMC4261878; doi:10.1186/1748-5908-9-86)
Supplement: Supplementary file 3 — Additional file 3: Analysis of covariance for secondary outcomes of change in attitude, subjective norm, and perceived behavioral control. (DOCX 85 KB) [file 13012_2013_796_MOESM3_ESM.docx]

**Supplementary File 3**

Analysis of covariance for secondary outcomes of change in attitude, subjective norm, and perceived behavioural control

|  |  |  |  |  |  | 95% CI | |
| --- | --- | --- | --- | --- | --- | --- | --- |
| TPB Construct | Source | *df* | *F* | *p* | B | Lower | Upper |
| Attitude | Covariate |  |  |  |  |  |  |
|  | ATT Pre-Intervention | 1 | 137.23 | 0.000 | 0.517 | 0.430 | 0.603 |
|  | Main Effects |  |  |  |  |  |  |
|  | Insert | 1 | 0.27 | 0.602 | 0.048 | -0.132 | 0.227 |
|  | Outsert | 1 | 0.39 | 0.534 | -0.069 | -0.288 | 0.149 |
|  | Reminder Note | 1 | 0.64 | 0.425 | -0.089 | -0.308 | 0.130 |
|  | Error | 432 |  |  |  |  |  |
| Subjective Norm | Covariate |  |  |  |  |  |  |
|  | SN Pre-Intervention | 1 | 69.54 | 0.000 | 0.370 | 0.283 | 0.457 |
|  | Main Effects |  |  |  |  |  |  |
|  | Insert | 1 | 5.03 | 0.025 | -0.251 | -0.472 | -0.031 |
|  | Outsert | 1 | 0.22 | 0.640 | 0.064 | -0.205 | 0.333 |
|  | Reminder Note | 1 | 0.29 | 0.593 | -0.073 | -0.343 | 0.196 |
|  | Error | 432 |  |  |  |  |  |
| Perceived Behavioural Control | Covariate |  |  |  |  |  |  |
|  | PBC Pre-Intervention | 1 | 117.97 | 0.000 | 0.436 | 0.357 | 0.514 |
|  | Main Effects |  |  |  |  |  |  |
|  | Insert | 1 | 1.48 | 0.225 | -0.113 | -0.296 | 0.070 |
|  | Outsert | 1 | 0.14 | 0.712 | 0.042 | -0.180 | 0.264 |
|  | Reminder Note | 1 | 0.31 | 0.578 | -0.063 | -0.286 | 0.160 |
|  | Error | 432 |  |  |  |  |  |
| *Note.* ATT = Attitudes; SN = Subjective Norms; PBC = Perceived Behavioural Control. | | | | | | | |
